# Lactoferrin Is an Allosteric Enhancer of the Proteolytic Activity of Cathepsin G

**DOI:** 10.1371/journal.pone.0151509

**Published:** 2016-03-17

**Authors:** Steffen Eipper, Robin Steiner, Adam Lesner, Marcin Sienczyk, David Palesch, Marc-Eric Halatsch, Ewa Zaczynska, Christopher Heim, Marcus D. Hartmann, Michal Zimecki, Christian Rainer Wirtz, Timo Burster

**Affiliations:** 1 Department of Neurosurgery, Ulm University Medical Centre, Ulm, Germany; 2 Faculty of Chemistry, University of Gdansk, Gdansk, Poland; 3 Faculty of Chemistry, Wroclaw University of Technology, Wroclaw, Poland; 4 Institute of Molecular Virology, Ulm University Medical Centre, Ulm, Germany; 5 Ludwik Hirszfeld Institute of Immunology and Experimental Therapy, Polish Academy of Sciences, Wroclaw, Poland; 6 Department of Protein Evolution, Max-Planck-Institute for Developmental Biology, Tübingen, Germany; CDFD, INDIA

## Abstract

Protease-mediated degradation of proteins is critical in a plethora of physiological processes. Neutrophils secrete serine proteases including cathepsin G (CatG), neutrophile elastase (NE), and proteinase 3 (PR3) together with lactoferrin (LF) as a first cellular immune response against pathogens. Here, we demonstrate that LF increases the catalytic activity of CatG at physiological concentration, with its highest enhancing capacity under acidic (pH 5.0) conditions, and broadens the substrate selectivity of CatG. On a functional level, the enzymatic activity of CatG was increased in the presence of LF in granulocyte-derived supernatant. Furthermore, LF enhanced CatG-induced activation of platelets as determined by cell surface expression of CD62P. Consequently, LF-mediated enhancement of CatG activity might promote innate immunity during acute inflammation.

## Introduction

Polymorphonuclear neutrophils harbour serine proteases including cathepsin G (CatG), neutrophile elastase (NE), and proteinase 3 (PR3) which are responsible for defense functions and for several physiological processes [1]. Nevertheless, neutrophils can also function in cathepsin G-deficient mice [2], most probably due to redundant activities of other serine proteases and biologically important proteins constituting the innate immune system.

In neutrophils, CatG, NE, and PR3 are stored in primary (azurophil) granules. After degranulation, these proteases are released at the site of inflammation to induce an innate and adaptive immune response [3, 4]. The biological activity of CatG is broad and encompasses enhancement of anti-infectious functions in a proteolytic-dependent and -independent manner [5–7], clearance of internalized pathogens [8], apoptosis [9], and proteolytic modification of chemokines and cytokines [10, 11]. Interestingly, CatG lowers low-density lipoprotein blood level and reduces atherosclerosis [12]. The effects of CatG are not restricted to the innate immunity since a crucial role of CatG was also demonstrated for the antigen presentation pathway and the stimulation of antigen-specific immune response [13–15]. Several naturally occurring compounds and proteins restrain CatG activity, for instance, glycosaminoglycans [16], and α1-antitrypsin [17]. However, when regulation of CatG activity fails, CatG is involved in pathophysiological processes. Excessive CatG function may lead to an impairment of defense processes to pathogens [18], autoimmunity [15, 19], or tumor progression [20].

The iron-binding LF of the transferrin family is stored in a separate compartment, the secondary granules of neutrophils, and is also secreted during inflammation [1, 21]. Apart from important functions associated with transport of iron, LF is a crucial mediator which supports an immune response by linking innate and adaptive immunity and maintaining immune homeostasis [22–24]. During inflammation the amount of granulocyte-derived lactoferrin (LF) can increase up to 200 μg/ml [25] and even higher in pulmonary secretion 100–1000 μg/ml [26] modulating the immune processes [21]. Additionally, LF possesses serine protease activity to prevent bacterial invasion [27]. Furthermore, LF interferes with glioblastoma cell growth [28], plays a protective role in oxidative stress induced damage, and displays regulatory activity by enhancing expression of cyclooxygenase 1 and inhibition of inducible cyclooxygenase 2 [29].

The acidic milieu during acute inflammation in the extracellular space is not optimal for the enzymatic activity of CatG and the functional interplay between CatG and LF under such circumstances has not been investigated. Thus, we sought to evaluate whether LF affects the catalytic activity of CatG. We found that LF potently increases the activity of CatG at pH 7.4 and to an even higher extent at pH 5 as well as in granulocyte-derived supernatant. Furthermore, LF might induce a conformational change of CatG resulting in an advanced substrate selectivity at P1 position as determined by different activity-based probes. Lastly, combination of LF and CatG significantly elevated the expression of the activation marker CD62P on platelets. Based on these findings, we propose that LF and CatG act synergistically during secretion by granulocytes augmenting the process associated with defense and tissue repair.

## Materials and Methods

### Reagents and chemicals

Diphenyl(1-((S)-1-((4-oxo-4-((5-(5-((3aS,4S,6aR)-2-oxohexahydro-1H-thieno[3,4-d]imidazol-4-yl)pentanamido)pentyl)amino)butanoyl)-L-valyl)pyrrolidine-2-carboxamido)-2-phenylethyl) phosphonate (Bt-LC-Suc-Val-Pro-Phe^P^(OPh)_2_; Marcin Sienczyk 116, MARS116) [30], bis(4-methoxyphenyl) (1-((S)-1-((5-((3aS,4S,6aR)-3a,6a-dimethyl-2-oxohexahydro-1H-thieno[3,4-d]imidazol-4-yl)pentanoyl)-L-valyl)pyrrolidine-2-carboxamido)-3-methylbutyl)phosphonate (Bt-Val-Pro-Leu^P^(OPhOMe)_2_, MARS123, Renata Grzywa, Faculty of Chemistry, Wroclaw University of Technology, Wroclaw, Poland), diphenyl(1-((S)-1-((5-((3aS,4S,6aR)-3a,6a-dimethyl-2-oxohexahydro-1H-thieno[3,4-d]imidazol-4-yl)pentanoyl)-L-valyl)pyrrolidine-2-carboxamido)-2-methylpropyl)phosphonate (Bt-Val-Pro-Val^P^(Ph)_2_, MARS125) [31], human recombinant lactoferrin (LF) from chinese hamster ovary (CHO) expression system (Marian Kruzel, Dept. of Integrative Biology and Pharmacology, University of Texas, Houston, USA) [32], human recombinant LF from rice (MyBioSource, San Diego, CA, USA), heparin (Sigma-Aldrich, Taufkirchen, Germany), neutrophil-derived human cathepsin G (CatG; BioCentrum Ltd., Krakow, Poland), dimethyl sulfoxide (DMSO, Serva, Heidelberg, Germany), CatG inhibitor I (CatGinh.; Merck Millipore, Darmstadt, Germany), neutrolphil elastase (NE) and proteinase 3 (PR3; Elastin Products Company, Inc., Owensville, MO, USA), PBS+0.05% Tween20 (PBS+T; Life Technologies, Darmstadt, Germany and Serva, Heidelberg, Germany), streptavidin-horseradish peroxidase (Vectastain; Burlingame, CA, USA), enhanced chemiluminescence (ECL) detection kit (GE Healthcare, München, Germany), polyvinylidene fluoride (PVDF; GE Healthcare, Freiburg, Germany), recombinant human CatL (R&D Systems, Wiesbaden, Germany), or 5 μg/ml recombinant human CatS (Enzo Life Sciences GmbH, Lörrach, Germany), 4-(((S)-3-methyl-1-((S)-2-(((S)-1-((4-nitrophenyl)amino)-1-oxo-3-phenylpropan-2-yl)carbamoyl)pyrrolidin-1-yl)-1-oxobutan-2-yl)amino)-4-oxobutanoic acid (Suc-VPF-pNA) [33], phorbol 12-myristate 13-acetate (PMA).

### Active-site label

The active-site label was performed as described in [30]. In brief, human LF (250 μg/ml, 3.28 μM) or heparin (250 μg/ml) were incubated with 800 ng/ml (28.07 nM) CatG in 0.1M citrate pH 5.0 or PBS pH 7.4 in the presence of the activity-based probe MARS116 (2 μM) with or without CatG inhibitor (preincubated for 15 min) for 1 h at room temperature (RT). Additionally, MARS123 or MARS125 (2 μM) were incubated with 800 ng/ml CatG, NE, or PR3 at pH 7.4. Samples were resolved by 12% sodium dodecyl sulfate polyacrylamide gel electrophoresis (SDS-PAGE) and protein contents were transferred to a PVDF membrane. The PVDF membrane was washed three times with PBS+T for 5 min at RT, incubated with Vectastain for 1h at RT, and followed by five additional wash steps. Protease activity was revealed using an ECL-detection kit and visualized using Hyperfilm ECL (GE Healthcare, München, Germany).

### *In vitro* processing of LF

50 μg/ml LF was incubated with 50 μg/ml CatG, 5 μg/ml recombinant CatL, or 5 μg/ml recombinant CatS for 6 h at 37°C in reaction buffer (0.1 M citrate, pH 5 for CatL or PBS, pH 7.4 for CatG or CatS). The digestion pattern was resolved by SDS-PAGE, stained with Coomassie blue (Life Technologies GmbH, Darmstadt, Germany) for 1 h, and destained with dest. water.

### Determination of CatG activity *via* colorimetric substrate

Kinetic measurement of CatG activity was accomplished by using the colorimetric substrate Suc-VPF-pNA. CatG (800 ng/ml), CatG with CatGinh. (10 μM), CatG with LF (250 μg/ml), CatG with CatGinh. and LF, or LF were preincubated for 15 min in PBS pH 7.4 at RT and followed by adding Suc-VPF-pNA (200 μM) [33]. The enzyme assay was performed in duplicates at 37°C, and absorption was determined at 405 nm (absorbance microplate reader, Bio-Rad, Model 550, Hercules, USA).

### Generating granulocyte-derived supernatant

Human blood was separated by gradient centrifugation in Gradisol G with a density of 1.115 g/ml (Aqua Medica, Poznan, Poland). Blood was over-layered to Gradisol and centrifuged at 400 x g for 30 min at RT. The granulocyte-rich layer was harvested by a hypotonic shock to remove erythrocyte contamination (H_2_O for 30 s followed by addition of 9 volumes of 10x Hanks’ solution). Cells were washed twice with Hanks’ solution and resuspended at density of 2 x 10^7^ cells/ml. The granulocytes were subsequently incubated for 60 min with 2 mg/ml of Zymosan (Sigma-Aldrich, Taufkirchen, Germany) at 37°C, centrifuged twice (20,000 x g, 30 min, 4°C), the supernatant was passed through a 0.22 μm filter, and frozen at -80° until use [34]. For PMA stimulation, cells were resuspended in Hanks’ solution at concentration of 5 x 10^6^/ml and incubated for 1h with 100 ng/ml PMA at 37°C in the absence of CO_2_ atmosphere. Afterwards the cell suspension was centrifuged twice (20,000 x g, 15 min, 4°C). The supernatant was taken, filtered through a 0.22 μm filter, and frozen at -80°C [35]. Zymosan was prepared according to the manufactures protocol (Sigma-Aldrich, Taufkirchen, Germany). In brief, 1% Zymosan suspension was boiled for 1h and centrifuged (20,000 x g, 30 min, 4°C). The Zymosan particles were opsonized by incubation for 30 min at 37°C (5 mg of Zymosan/ml of human serum) followed by two wash steps (20,000 x g, 30 min, 4°C) with Hanks’ solution. Granulocyte-derived supernatant was incubated with or without 250 μg/ml LF (MyBioSource, San Diego, CA, USA) and active CatG was determined by using MARS116 as described above. For Western blot analysis, the same PVDF membrane was incubated with anti-human LF antibody (0.1 μg/ml; eBioscience, San Diego, CA, USA), washed with PBS+T for three times (each 5–10 min), and secondary antibody (anti-mouse-HRP, Santa Cruz Biotechnology, Dallas, TX, USA) was added for 1 h at RT. After three wash steps, levels of LF were visualized by ECL and x-ray film. Use of human cells from buffy coats for *in vitro* studies was in accordance with IRB regulations. The blood donors provided their written consent to the DRK-Blutspendezentrale that their blood could be used for scientific studies. The Ethikkommission of the Universität Ulm, application No. # 327/14, approved this study.

### Platelet activation assay

Cells from buffy coats were washed with PBS pH 7.4 for three times (centrifugation, 317 x g, 5 min, RT) and seeded on a 96-well plate (1.7x10^7^ cells/ml per well in 100 μl PBS). CatG (8, 4, 2, or 1 μg/ml) with or without LF 250 μg/ml (human recombinant LF from rice), LF and CatG with CatG inhibitor (10 μM in 10% DMSO), LF and CatG with DMSO (10%), or LF was added and incubated for 30 min at RT. Afterwards, cells were washed twice in PBS containing 1% FBS (402 x g, 5 min, 4°C) and stained with CD42b-PE and CD62P-APC, (Biolegend, San Diego, CA, USA) diluted in PBS containing 1% FBS and kept on ice for 30 min. Cells were washed twice (390 x g, 5 min, 4°C) and measured by flow cytometer (FACSCalibur; BD Biosciences, Franklin Lakes, NJ, USA). 7-AAD (Biolegend, San Diego, CA, USA) was added before analysis and FSC as well as SSC were set to log scale [36]. 100.000 cells were collected and the resulting data were analyzed by using FlowJo software (Tree Star Inc., Ashland, OR, USA).

### Statistical analysis

The density of the active-site label was assessed by using ImageJ 1.49 (NIH, Bethesda, MD, USA) and data were depicted as mean ± standard error of the mean (S.E.M.). Statistical analysis was performed using the unpaired, two-tailed Student’s *t*-test (Prism 4, GraphPad Software, La Jolla, CA, USA).

## Results

### Lactoferrin (LF) increases CatG activity

**Fig 1:**
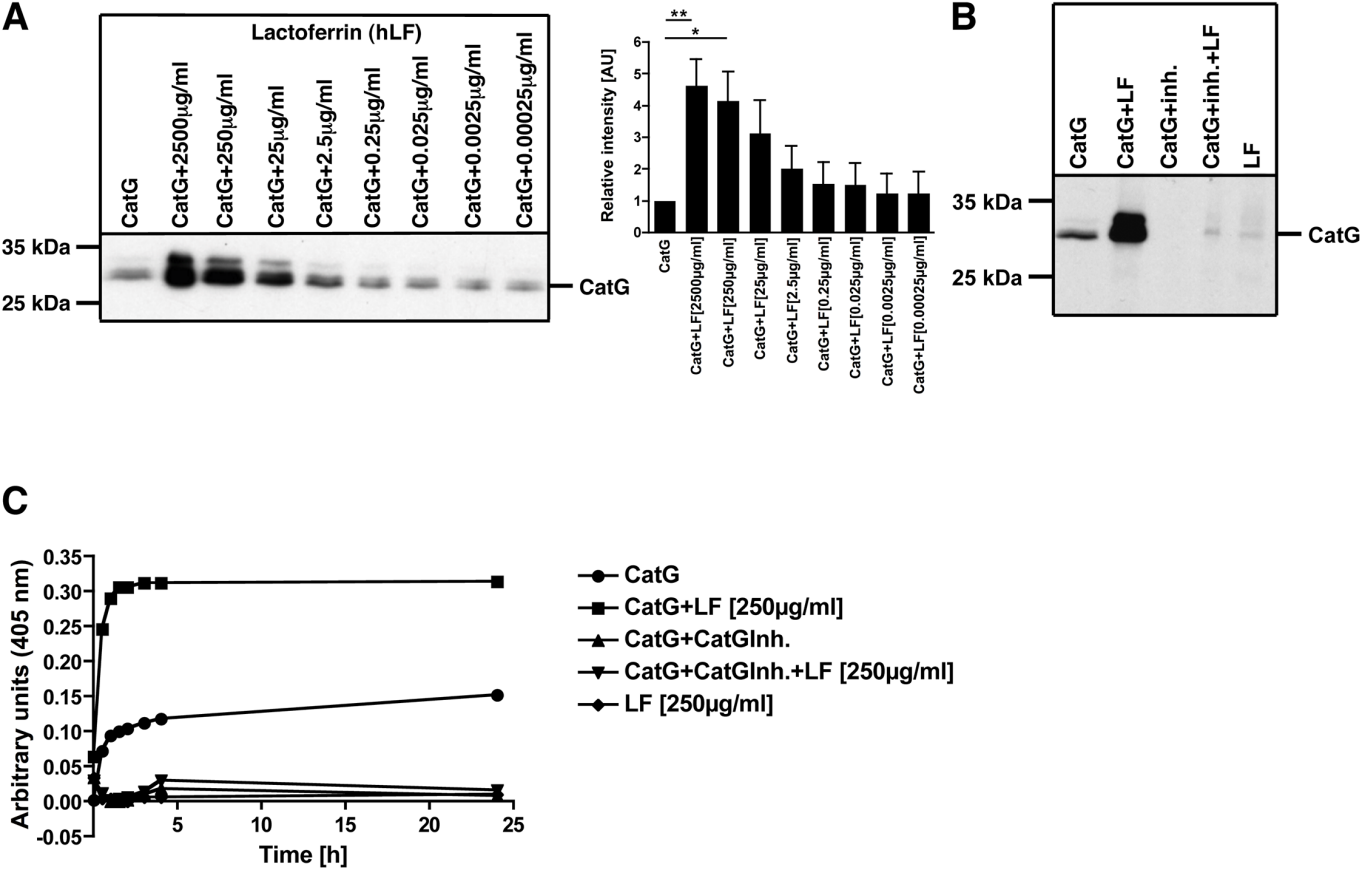
Determine of cathepsin G (CatG) activity in the presence of lactoferrin (LF). A) 800 ng/ml of purified human CatG were incubated with indicated amounts of LF together with the activity-based probe MARS116 for 1 h at RT. MARS116 binds covalently to the active site of CatG. The CatG-MARS116 complex was resolved by SDS-PAGE, transferred to PVDF membrane, and visualized by streptavidin-HRP (left panel). The density of the respective bands were quantified by ImageJ and summarized in the bar diagram (right panel), n = 5. B) 800 ng/ml (28.07 nM) of CatG, CatG with 250 μg/ml (3.28 μM) LF, CatG preincubated with CatG inhibitor (10μM), CatG preincubated with CatG inhibitor and then added LF, or LF were incubated with MARS116 for 1 h at RT. LF 3.28 μM:CatG 28.07 nM ratio = 116.85. Two independent experiments. C) CatG activity was analyzed by using the colorimetric substrate Suc-VPF-pNA. 800 ng/ml CatG, CatG with CatG inhibitor (10 μM), LF (250 μg/ml, 3.27 μM), CatG with CatG inhibitor and LF, or LF were preincubated for 15 min at RT. Afterwards, the Suc-VPF-pNA was added and the measurements were performed in duplicates. One representative diagram is shown (two independent experiments, n = 2).

LF has been reported to inhibit CatG [37]. Thus, in a first set of experiments, we tested whether LF diminishes CatG activity. To this end, CatG was incubated with different concentrations of LF and CatG activity was determined by using the activity-based probe MARS116. MARS116 forms a covalent bond to the active center of CatG and can be resolved by SDS-PAGE and visualized *via* streptavidin-HRP blot. Strikingly, LF enhances CatG activity at a minimum concentration of 2.5 μg/ml LF and up to 250 μg/ml LF (Fig 1A) which resembles physiological concentrations [25]. In a control experiment, LF augments CatG activity and was reduced to background levels by using the CatG inhibitor (Fig 1B). Moreover, LF alone was not visualized where CatG normally migrates on the gel (28.5 kDa) ruling out the possibility that LF might be detected by MARS116. LF-mediated enhancement of CatG activity was verified by applying the colorimetric substrate Suc-VPF-pNA. We found that the digestion of Suc-VPF-pNA was higher when LF was added to CatG in comparison to the sample which only contained CatG (Fig 1C). The CatG inhibitor served as a control and inhibited the turnover of the respective substrate. Thus, LF is a potent enhancer of CatG activity.

### CatG and NE do not proteolytically degrade LF molecules *in vitro*

**Fig 2:**
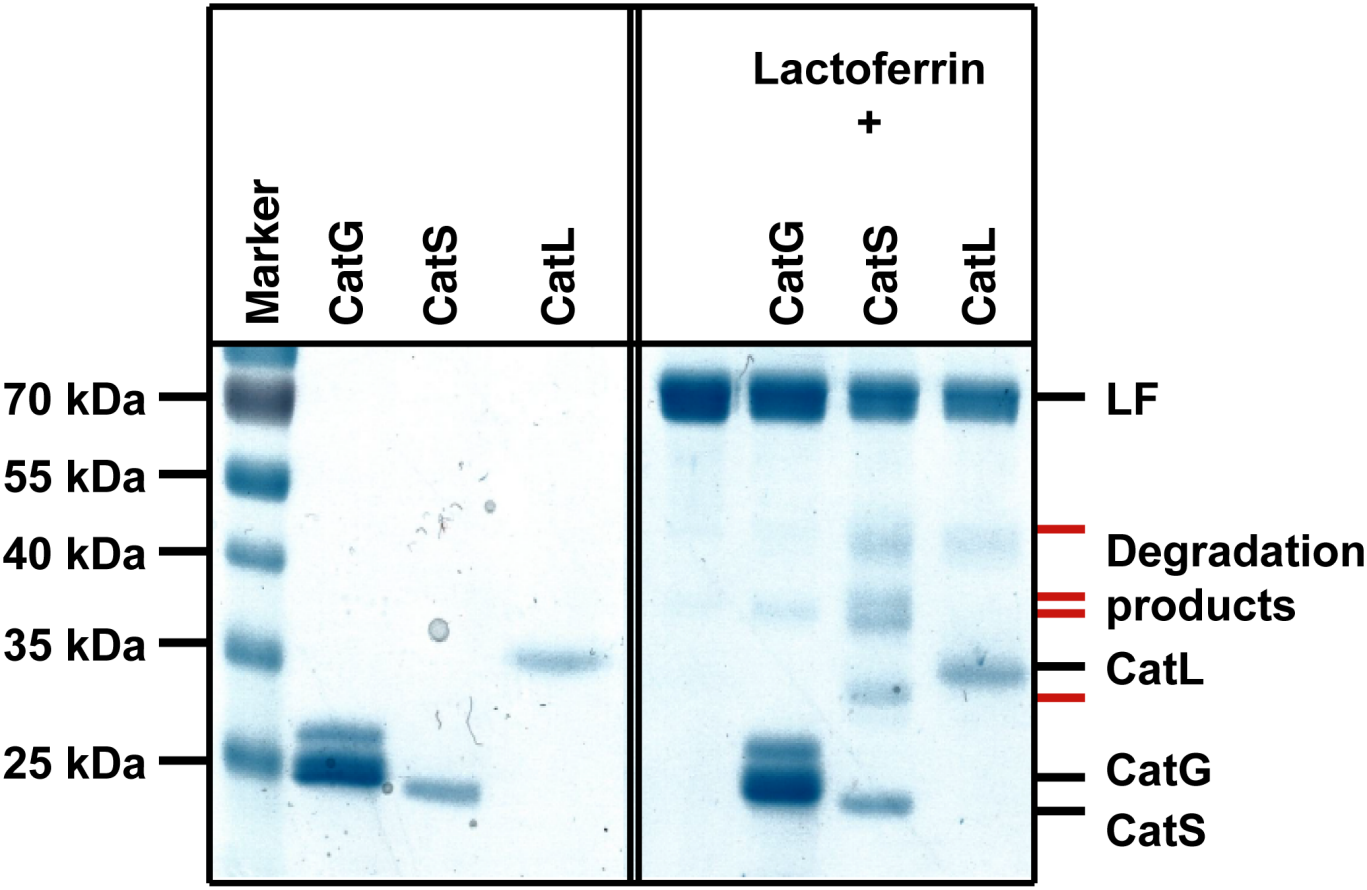
Degradation of LF. Recombinant human LF (50 μg/ml) was incubated with CatG (50 μg/ml) or CatS (5 μg/ml) at pH 7.4 and CatL (5 μg/ml) at pH 5.0 for 6 h at 37°C. The degradation products were visualized by SDS-PAGE and Coomassie staining. A representative result of three independent experiments is shown.

Previously, LF degradation was attributed to CatB, CatL, and CatS [38]. We examined LF processing to test whether LF is also a substrate for CatG. A panel of different human cathepsins were incubated with LF and the processing products were visualized by SDS-PAGE and Coomassie staining as demonstrated in Fig 2. As expected, CatL and CatS processed LF. However, CatG did not digest LF indicating that the measurement of higher CatG activity in Fig 1A was not due to any degradation artifacts which otherwise would be visualized by MARS116 and migrate around 28 kDa. Furthermore, this suggests that the CatG enhancing activity of LF is mediated by full length LF protein and not by a CatG-generated degradation product.

### LF stabilizes CatG

**Fig 3:**
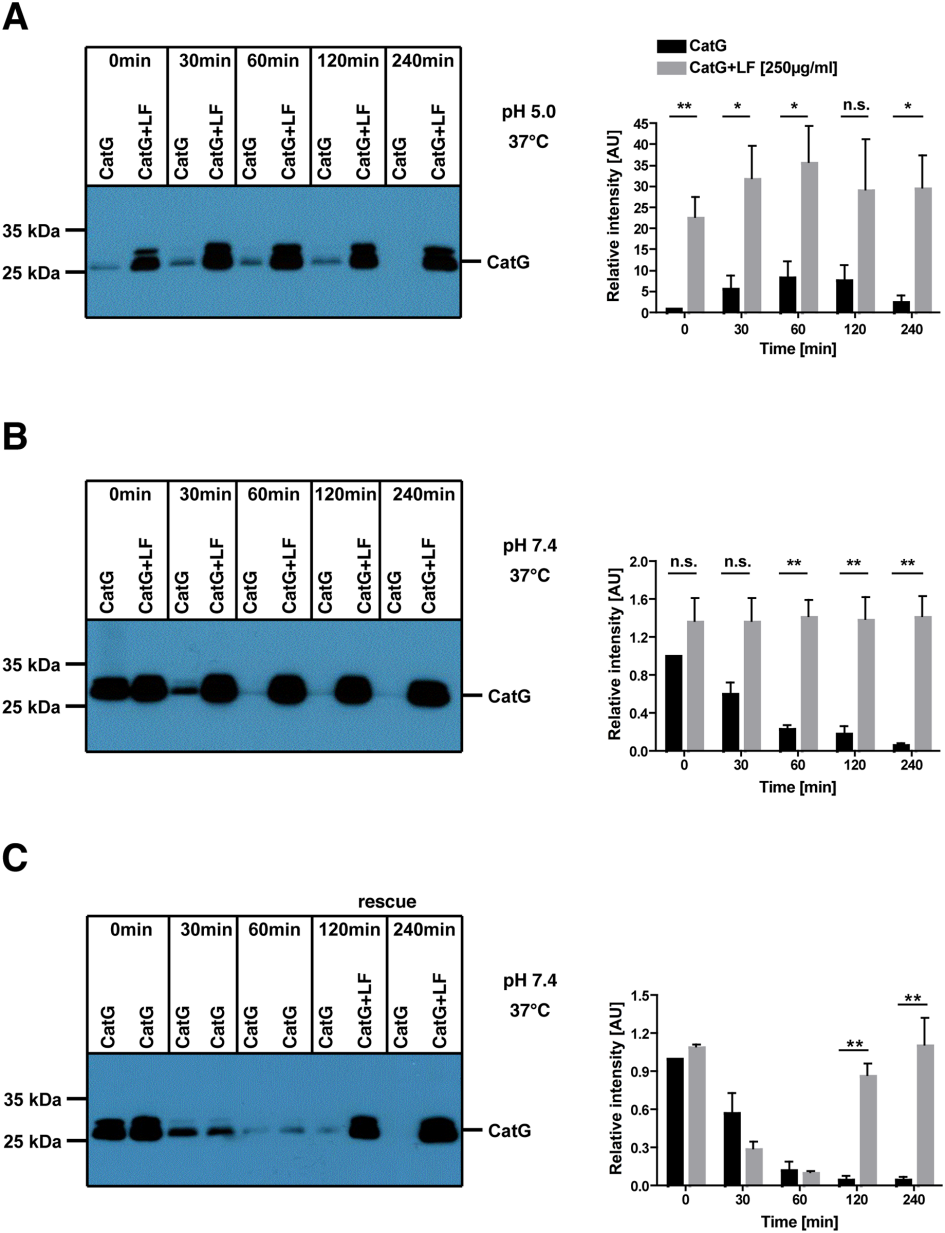
Analysis of pH dependent enhancement of CatG activity. CatG (800 ng/ml) or both LF (250 μg/ml, 3.27 μM) and CatG were incubated with MARS116 for the indicated time points under A) pH 5 or B) pH 7.4 conditions at 37°C. Samples were resolved by SDS-PAGE, transferred to PVDF membrane, and visualized by streptavidin-HRP. C) CatG and MARS116 were independently preincubated for 0, 30, or 60 min. After 60 min, in one set of samples LF was added and further incubated. The band intensity was analyzed by ImageJ and summarized (right panel). Three independent experiments were performed.

Local acidosis predominates at the site of inflammation. Thus, we sought to investigate whether LF-mediated enhancement of CatG activity is pH dependent. To this end, we incubated CatG under pH 5 or pH 7.4 conditions and analyzed CatG activity at different time points. LF exhibited a striking increase in CatG activity at pH 5 compared to pH 7.4. The effect was independent from the incubation time (Fig 3A and 3B). This suggests that LF maintains the catalytic activity of CatG at pH 5 since CatG has its highest activity under neutral conditions. In a CatG rescue experiment, CatG was preincubated for 30 min or 60 min and then LF was added to CatG for a further incubation time of 60 min or 180 min, respectively. Indeed, LF rescued CatG activity compared to the control CatG (Fig 3C) providing more evidence for the model that LF stabilizes CatG.

### CatG changes substrate specificity under the influence of LF

**Fig 4:**
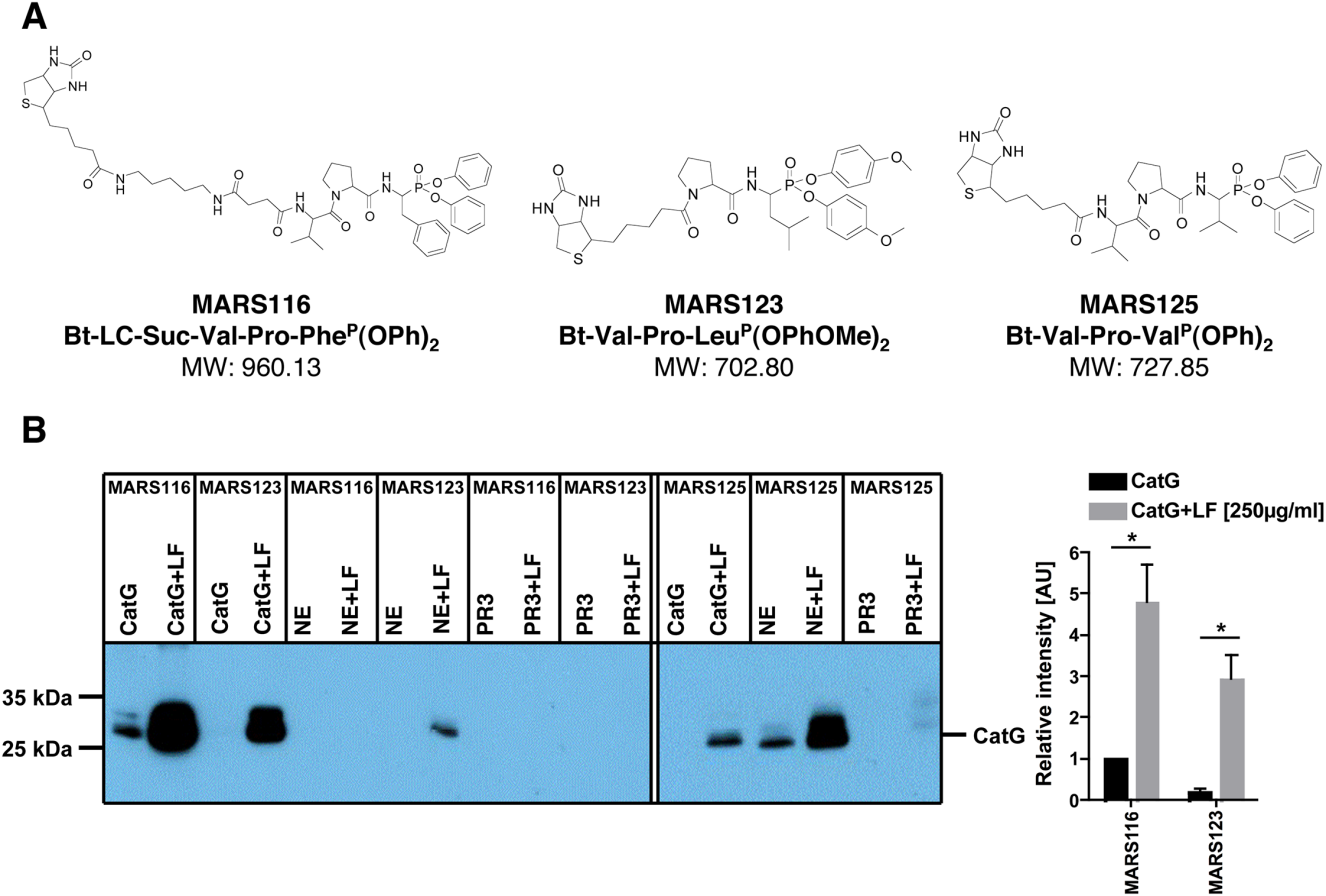
Use of different activity-based probes to visualize CatG activity. A) The structure of MARS116, MARS123, and MARS125. B) 800 ng/ml of CatG, NE, or PR3 with or without 250 μg/ml (3.27 μM) LF were quantified by using MARS116, MARS123, or MARS125. One representative out of three independent experiments is shown (left panel). The CatG band intensity was quantified and summarized in the bar diagram (right panel).

Next, we sought to determine whether LF might direct the active site of CatG towards a more open conformation thereby losing its substrate specificity. To elucidate this in more detail, different activity-based probes were incubated with CatG. MARS123, which harbors the amino acid leucine (Leu) at P1 position, and MARS125, with a valine (Val) moiety at P1 position (Fig 4A), were not able to covalently bind to the active center of CatG (Fig 4B). However, by adding LF to the assay CatG activity was visualized with both MARS123 and MARS125. Furthermore, NE and PR3 were treated with MARS116, MARS123, or MARS125 and proteases were visualized according to their catalytic mechanism. LF boosted also NE-activity, but PR3-activity did not change in the presence of LF. LF increases the activity of the two major proteases secreted by neutrophils, CatG and NE. Taken together, CatG loses its substrate specificity in the presence of LF.

### Heparin does not aggravate CatG activity

**Fig 5:**
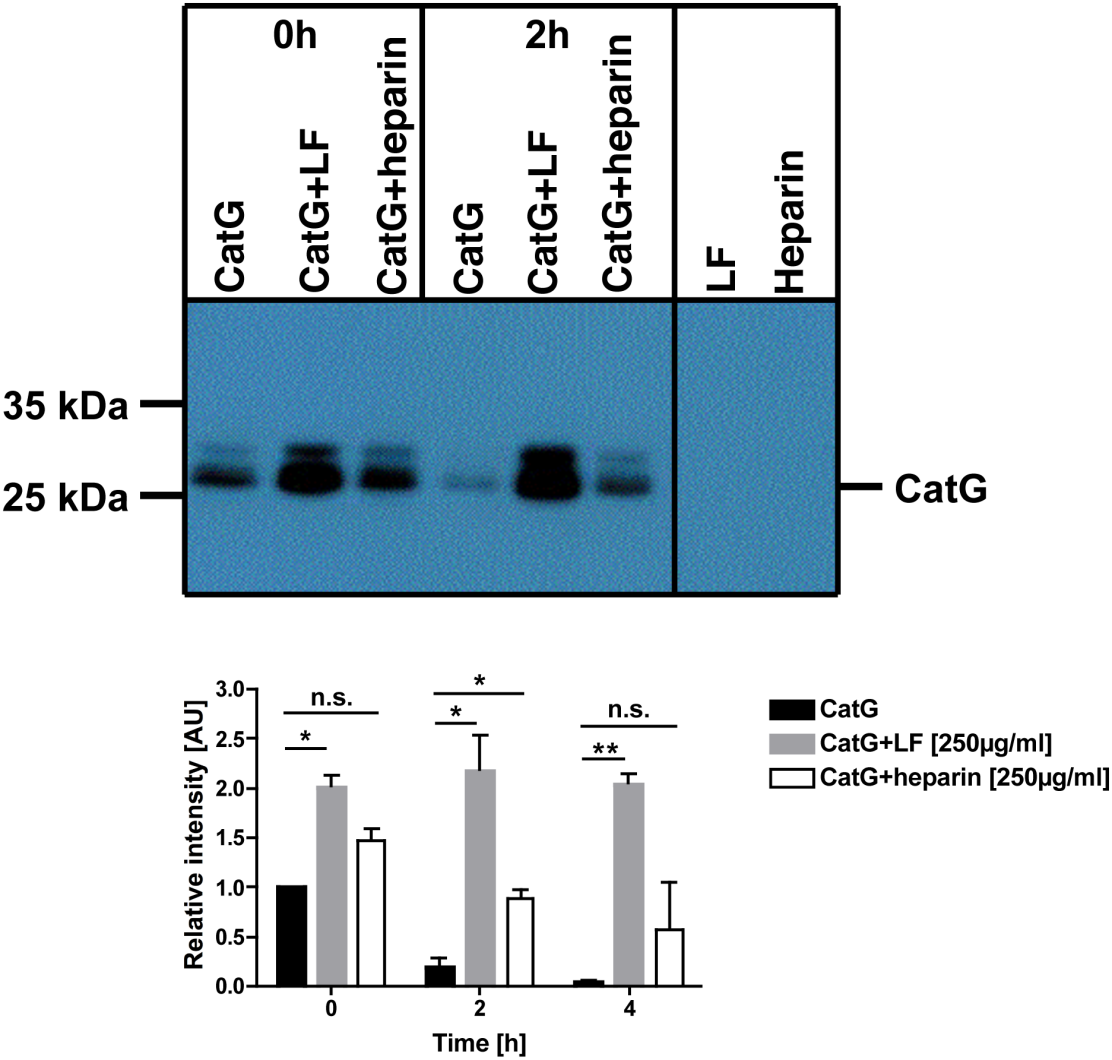
Detection of CatG activity under the control of LF or heparin. 800 ng/ml CatG was incubated with or without LF or heparin (250 μg/ml) and MARS116 for 0 h or 2 h. CatG activity was detected via SDS-PAGE and streptavidin-HRP blot (upper panel). One representative out of two independent experiments is shown. The intensity of the respective bands were determined by ImageJ (lower panel).

CatG, like LF, is a basic protein, due to its positively charged amino acids. CatG has a high electrostatic affinity to heparin, since heparin is a proteoglycan and harbors an anionic charged moiety [39, 40]. Further experiments were performed to elucidate the involvement of heparin in regulating CatG activity. Hence, CatG was treated with either LF or heparin for the indicated time points, and we found that also heparin provoked a significantly increased binding of CatG to MARS116 (Fig 5). This gives rise to the speculation that a negatively charged proteoglycan like heparin does increase CatG activity.

### LF increases the CatG activity in granulocyte supernatant

**Fig 6:**
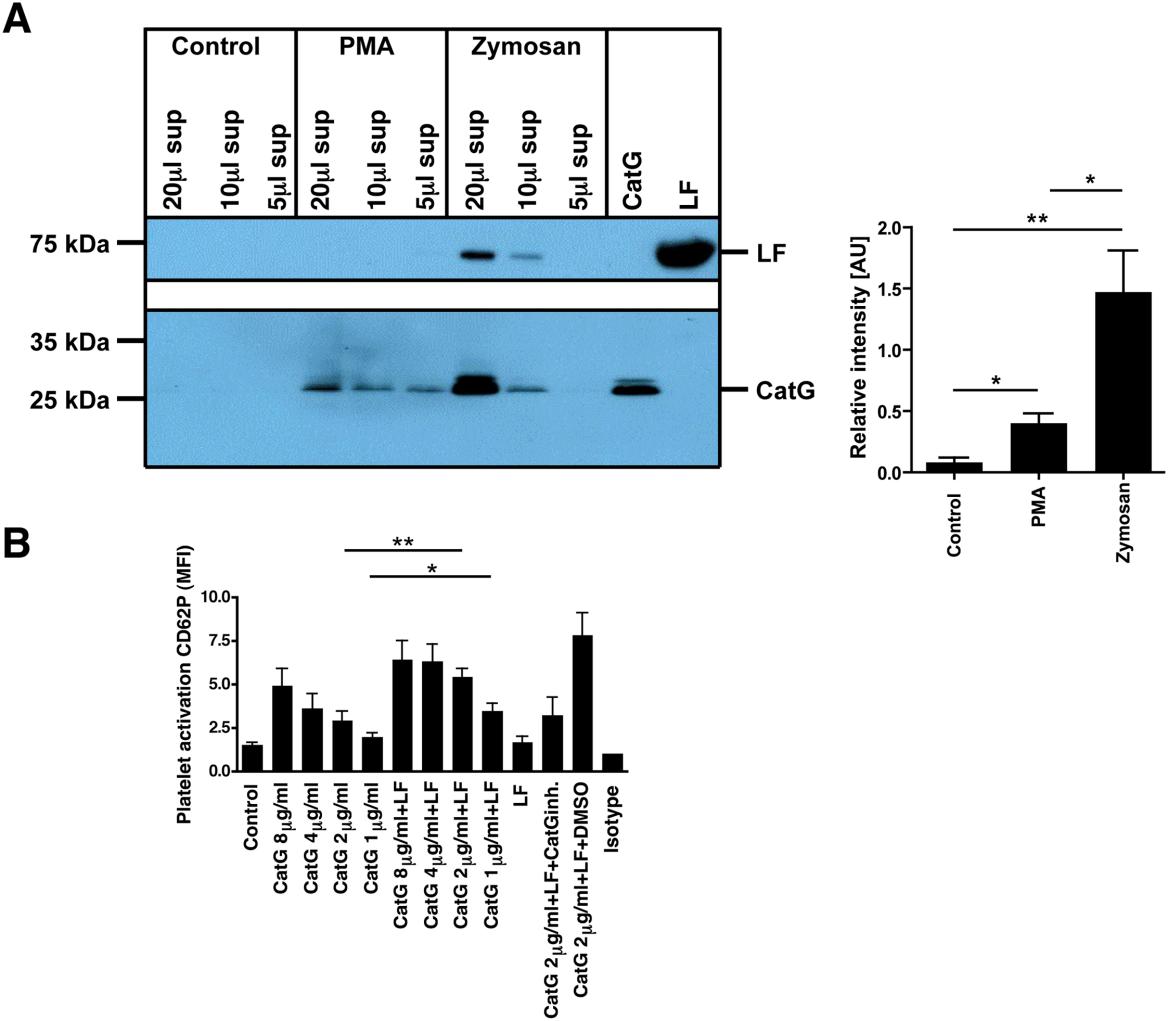
Functional assay to determine physiological relevance of LF-mediated enhancement of CatG activity. A) Granulocytes were treated with PMA, Zymosan, or kept in medium (control). The granulocyte-derived supernatant was incubated with MARS116 and CatG activity was detected after SDS-PAGE and streptavidin-HRP blot. LF was visualized by LF-specific immunoblot (left panel). The CatG band intensity was quantified by ImageJ and summarized (right panel). One representative out of two independent experiments is shown. B) Human blood from buffy coats were incubated with CatG (8, 4, 2, or 1 μg/ml), with or without LF (250 μg/ml, 3.27 μM), CatG with CatG inhibitor (10 μM) and LF, or CatG and LF with DMSO for 30 min at RT. Platelet activation was determined by the increase of cell surface levels of CD62P analyzed by flow cytometry (n = 5 different donors; for CatG+LF+CatGinh. and CatG+LF+DMSO, n = 3).

Both, primary and secondary granules which harbor CatG or LF, respectively, are secreted by neutrophils at the site of inflammation [4]. To test whether LF and CatG are secreted and CatG activity is thereby increased in granulocyte-derived supernatant, granulocytes were isolated from human blood and stimulated with either PMA or Zymosan to trigger degranulation. CatG activity was determined by using MARS116 and the LF content was analyzed by LF-specific immunoblot. Fig 6A demonstrates that CatG activity is indeed higher in Zymosan-stimulated granulocyte-supernatant, in which LF and CatG activity were detected compared to control- or PMA stimulated granulocytes. In a further experiment, we added LF to granulocyte-derived supernatant and found that CatG activity was detected in control samples when LF was spiked to the respective supernatant. In the supernatant of PMA-treated granulocytes CatG activity was enhanced by LF but not in supernatant from Zymosan-treated granulocytes (S1 Fig). Consequently, activation of granulocytes by Zymosan to simulate inflammation revealed that CatG activity was higher when LF was present.

To explore the CatG-mediated activation of platelets, human blood was treated with different concentrations of CatG [41] in the presence or absence of LF and the activation of platelets was determined by CD62P which is an activation marker of platelets. The cells were gated according to a previous publication [36] (S2 Fig). CatG increased the activation of platelets in a concentration-dependent manner and activation was further enhanced by adding LF to the assay compared to only LF which had no effect (Fig 6B). The abundant CatG activity was diminished by the use of the CatG inhibitor which decreases platelet activation to background levels and thereby indicates that the effect is due to CatG activity. In conclusion, LF supports a CatG-mediated immune response of granulocytes.

## Discussion

LF and CatG are crucial mediators of the immune response during acute inflammation. Even though it was previously published that LF inhibits CatG activity and might limit hypersensitivity reaction (allergy) as well as protease-induced inflammation [37]; however, one would assume that LF and CatG should act additively or even synergistically over the course of an immune response. Indeed, LF does not degrade CatG and vice versa, which might not account for an inhibitory capacity by the proteolytic action of LF or CatG. In addition to our enzymatic assay data, we measured CatG activity with a panel of activity-based probes and clearly showed LF-mediated enhancement of CatG activity. Furthermore, co-treatment of CatG with LF facilitates CatG binding to MARS125, which harbors a valine at P1 position and is generally not a substrate for CatG. This mechanism might be critical in maintaining intracellular (phagolysosome) as well as extracellular degradation of pathogen-derived proteins due to the toleration of escape mutations.

In activated neutrophils, granules are mobilized and fuse with phagosomes where both CatG and LF meet [42] or these granules traffic to the plasma membrane where their content is secreted to the site of inflammation [8]. Bacteria, on the other hand, create an acidic microenvironment by anaerobic metabolism [43] which might decrease the performance of CatG, since its pH optimum is neutral. However, the presence of LF increases the catalytic activity of CatG, reduces substrate selectivity, and might compensate for a low pH at the site of inflammation. Moreover, we found that LF further enhanced CatG-induced activation of platelets in a proteolytic-dependent manner which might be crucial in wound healing and immunity. LF is a critical mediator to promote the proteolytic performance of CatG during an immune response

## Supporting Information

S1 Fig Detection of CatG activity in LF spiked granulocyte-derived supernatant. The indicated granulocyte-derived supernatant (control, PMA, or zymosan) was incubated with or without LF (250 μg/ml) and MARS116. CatG activity was determined by SDS-PAGE and streptavidin-HRP blot. On the same PVDF membrane, LF was detected by using LF-specific antibody. Sup = supernatant.
(PPT)

S2 Fig Gating strategy of platelet activation assay. Cells were incubated with CatG or the combination of CatG and LF. The platelet cell population was determined by CD42b (upper, left panel), and the activation status of platelets was analyzed by CD62P (lower panel).
(PPT)

## Abbreviations

Cat: cathepsin
CatGinh: cathepsin G inhibitor
DMSO: dimethyl sulfoxide
LF: lactoferrin
MARS: Marcin Sienczyk
NE: neutrophile elastase
PMA: phorbol 12-myristate 13-acetate
PR3: proteinase 3
PVDF: polyvinylidene fluoride
RT: room temperature
Suc-VPF-pNA: 4-(((S)-3-methyl-1-((S)-2-(((S)-1-((4-nitrophenyl)amino)-1-oxo-3-phenylpropan-2-yl)carbamoyl)pyrrolidin-1-yl)-1-oxobutan-2-yl)amino)-4-oxobutanoic acid
sup: supernatant
