# Supplementary figures and images for: Lactoferrin Is an Allosteric Enhancer of the Proteolytic Activity of Cathepsin G
Source: PLoS One. 2016 Mar 17;11(3):e0151509. doi: 10.1371/journal.pone.0151509 (PMC4795699; doi:10.1371/journal.pone.0151509)

## Slide 1
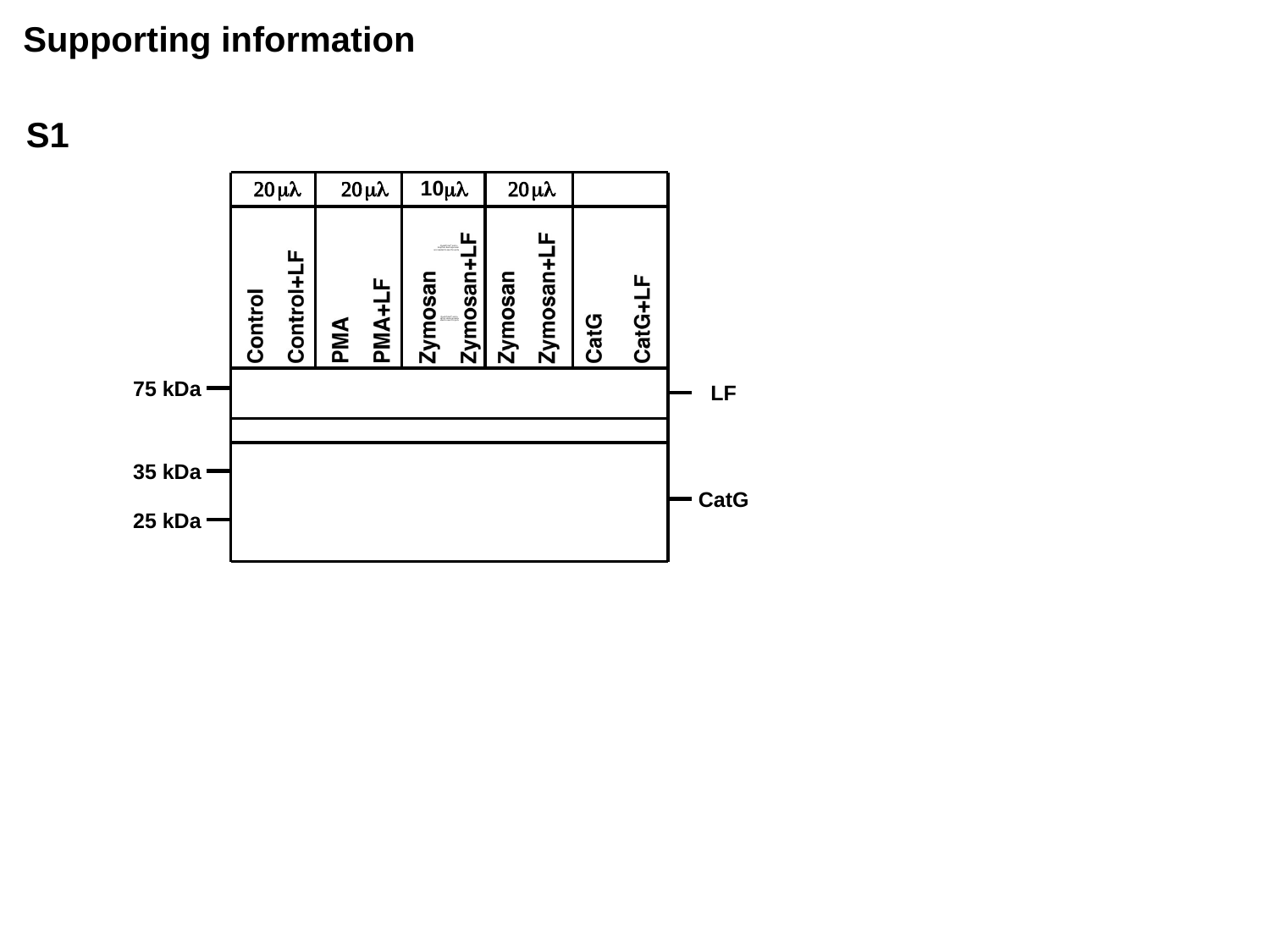

Supporting information
S1

Supplement: S1 Fig — The indicated granulocyte-derived supernatant (control, PMA, or zymosan) was incubated with or without LF (250 μg/ml) and MARS116. CatG activity was determined by SDS-PAGE and streptavidin-HRP blot. On the same PVDF membrane, LF was detected by using LF-specific antibody. Sup = supernatant. (PPT) [file pone.0151509.s001.ppt]

## Slide 1
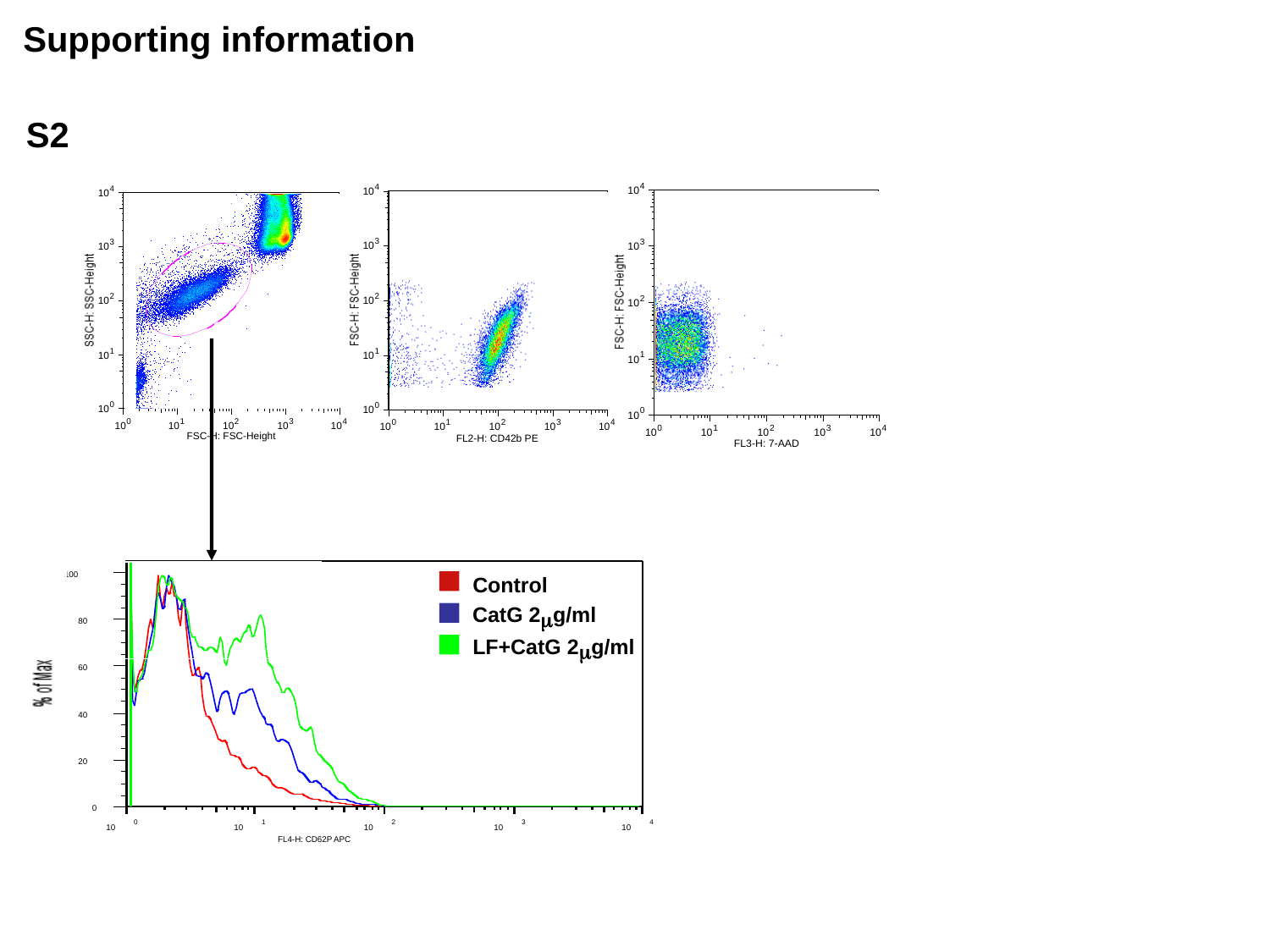

Supporting information
S2
Control
CatG 2
g/ml

LF+CatG 2
g/ml


Supplement: S2 Fig — Cells were incubated with CatG or the combination of CatG and LF. The platelet cell population was determined by CD42b (upper, left panel), and the activation status of platelets was analyzed by CD62P (lower panel). (PPT) [file pone.0151509.s002.ppt]
